# *STAT6*, *PBX2*, and *PBRM1* Emerge as Predicted Regulators of 452 Differentially Expressed Genes Associated With Puberty in Brahman Heifers

**DOI:** 10.3389/fgene.2018.00087

**Published:** 2018-03-20

**Authors:** Loan T. Nguyen, Antonio Reverter, Angela Cánovas, Bronwyn Venus, Stephen T. Anderson, Alma Islas-Trejo, Marina M. Dias, Natalie F. Crawford, Sigrid A. Lehnert, Juan F. Medrano, Milt G. Thomas, Stephen S. Moore, Marina R. S. Fortes

**Affiliations:** ^1^School of Chemistry and Molecular Biosciences, The University of Queensland, St. Lucia, QLD, Australia; ^2^Faculty of Biotechnology, Vietnam National University of Agriculture, Hanoi, Vietnam; ^3^CSIRO Agriculture and Food, Queensland Bioscience Precinct, St. Lucia, QLD, Australia; ^4^Centre for Genetic Improvement of Livestock, Department of Animal Biosciences, University of Guelph, Guelph, ON, Canada; ^5^Queensland Alliance for Agriculture and Food Innovation, The University of Queensland, St. Lucia, QLD, Australia; ^6^School of Biomedical Sciences, The University of Queensland, Brisbane, QLD, Australia; ^7^Department of Animal Science, University of California, Davis, Davis, CA, United States; ^8^Departamento de Zootecnia, Faculdade de Ciências Agráìrias e Veterináìrias, Universidade Estadual Paulista Júlio de Mesquita Filho, São Paulo, Brazil; ^9^Department of Animal Science, Colorado State University, Fort Collins, CO, United States

**Keywords:** *Bos indicus*, puberty, gene expression, RNA sequencing, gene network, liver

## Abstract

The liver plays a central role in metabolism and produces important hormones. Hepatic estrogen receptors and the release of insulin-like growth factor 1 (IGF1) are critical links between liver function and the reproductive system. However, the role of liver in pubertal development is not fully understood. To explore this question, we applied transcriptomic analyses to liver samples of pre- and post-pubertal Brahman heifers and identified differentially expressed (DE) genes and genes encoding transcription factors (TFs). Differential expression of genes suggests potential biological mechanisms and pathways linking liver function to puberty. The analyses identified 452 DE genes and 82 TF with significant contribution to differential gene expression by using a regulatory impact factor metric. Brain-derived neurotrophic factor was observed as the most down-regulated gene (*P* = 0.003) in post-pubertal heifers and we propose this gene influences pubertal development in Brahman heifers. Additionally, co-expression network analysis provided evidence for three TF as key regulators of liver function during pubertal development: the signal transducer and activator of transcription 6, PBX homeobox 2, and polybromo 1. Pathway enrichment analysis identified transforming growth factor-beta and Wnt signaling pathways as significant annotation terms for the list of DE genes and TF in the co-expression network. Molecular information regarding genes and pathways described in this work are important to further our understanding of puberty onset in Brahman heifers.

## Introduction

The beef industry in Northern Australia is facing increased demand for improved herd productivity. Brahman cattle, a breed of the *Bos indicus* sub-species, can withstand hot-humid conditions, but enter puberty at older age in comparison with *Bos taurus* (Johnston et al., 2009). Late onset of puberty in *B. indicus* predicts a decrease in lifetime productivity (Lesmeister et al., 1973; Johnston et al., 2013). Therefore, reducing the age at puberty to increase *B. indicus* cow productivity is a worthwhile goal for management and breeding.

Reproduction is an energy intensive process that is likely to require specific involvement of the liver. The physiological mechanisms controlling energy balance are closely linked to fertility, to minimize the risk that pregnancy and lactation coincide with periods of nutritional stress (Mircea et al., 2007). While in placental mammals, the hypothalamus–pituitary–ovaries axis takes precedence in the integration of metabolic and reproductive status, there are evidences for the involvement of the liver in this process. In the postpartum cow, it is known that the metabolic stress associated with transition is linked to impaired liver function and delayed ovulation (Montagner et al., 2016). In mice, it has been shown that hepatic synthesis of Insulin-like growth factor 1 (IGF1) is regulated by amino acid-dependent activation of ERα in the liver (Della Torre et al., 2011). Additionally, an association between single nucleotide polymorphisms in genes of the IGF1 signaling pathways and age at puberty in Brahman cattle was observed (Fortes et al., 2013).

Several studies investigated the change in hepatic mRNA expression of genes encoding proteins that participate in various processes including growth hormone signaling, liver lipoprotein assembly, ureagenesis, and gluconeogenesis (Loor, 2010). Few studies have utilized microarray technology to evaluate hepatic metabolic adaptations to dairy cow throughout pregnancy, transition period, early lactation, and mid lactation (Herath et al., 2004; Loor et al., 2005; Loor, 2010; McCarthy et al., 2010; Akbar et al., 2013). A microarray study of the effect of pregnancy and diet in liver gene expression revealed specific hepatic adaptations of beef cows to different nutritional environments. For example, the study found clear evidence of gluconeogenesis in the liver of pregnant cows during limited forage availability (Laporta et al., 2014). Using candidate gene approach or transcriptomics investigating genomic peripartal adaptions in dairy cows provided insights into physiological function and genetics of key tissues.

A study has evaluated the liver transcriptome during puberty onset in Brangus heifers (3/8 Brahman; *B. indicus* × 5/8 Angus; *B. taurus*) (Cánovas et al., 2014). This study has used RNA sequencing, which is a more sensitive transcriptome profiling method than microarray. Sequencing RNA is capable of detecting not only expression differences in the most highly expressed metabolic genes, but also in regulatory genes (Marioni et al., 2008). Our study used sequencing to evaluated mRNA expression, regulatory factors, and potential biological pathways that occur in the liver related to pubertal development in Brahman heifers; a different population that is predominantly *B. indicus*. These heifers were used in two studies that reported transcriptomics of the hypothalamus–pituitary–ovarian axis, with no observation of liver function (Fortes et al., 2016; Nguyen et al., 2017). Molecular information of key regulators and pathways in liver may reveal mechanisms involved in puberty onset and energy metabolism. To access IGF1 signaling in this context, we also report hormonal measurements. This information may contribute to future approaches for reducing the age at puberty of *B. indicus* cattle used in tropical beef production systems.

## Materials and Methods

### Animals and Samples

Heifers used in this study were managed, handled, and euthanized as per approval of the Animal Ethics Committee of the University of Queensland, Production, and Companion Animal group (certificate number QAAFI/279/12). Heifer used were 12 young animals from commercial Queensland herds with typical phenotypic characteristics of *B. indicus* cattle. In Australia, the average content of *B. indicus* in Brahman cattle is about 95% (Porto Neto et al., 2013). They were unrelated heifers of similar age (born during the wet season 2011/2012) and weight <250 kg. They were maintained on pasture at the Gatton Campus facilities of the University of Queensland.

We performed ultrasound observations of pubertal development every fortnight from October 2012 to May 2013. With ultrasound, the observation of the first *corpus luteum* (CL) was used to define pubertal status (Johnston et al., 2009). Euthanasia occurred 15 days after the observation of the first CL, with samples collected in the next estrous cycle. Six post-pubertal heifers were euthanized during the luteal phase of their second estrous cycle, confirmed by the observation of the second CL at euthanasia. Serum progesterone concentrations were measured to confirm a functional CL in post-pubertal heifers (2.0 ± 0.7 ng/mL, mean ± SE). Pre-puberty heifers were randomly selected from the group that had never ovulated (plasma progesterone concentration 0.4 ± 0.2 ng/mL, mean ± SE) and paired with post-pubertal animals in slaughter day. Therefore, on each slaughter day, two heifers were euthanatized, one pre- and one post-puberty.

Serum progesterone concentrations were measured in hexane extracts by RIA (Curlewis et al., 1985) at the Laboratory for Animal Endocrinology at the University of Queensland. The extraction efficiency was 75% and reported values were not corrected for these losses. The assay sensitivity was 0.1 ng/mL and the within-assay coefficient of variation was 5.0%.

Circulating IGF1 concentrations were measured using a commercial radioimmunoassay kit (10IGF100 Kit; Bioclone, Sydney, NSW, Australia). The method included an acid–ethanol extraction to remove IGF1-binding proteins and measure total IGF1. All samples were analyzed within a single assay kit as previously described (Dahlanuddin et al., 2014). The assay sensitivity was 0.2 ng/mL and the within-assay coefficient of variation was 2.5%.

Body weight (BW, kg) and body condition scores (BCSs, 5-point scale) were also measured before tissue harvesting, as previously described (Fortes et al., 2016). BWs were 338 ± 54 and 363 ± 39 kg and BCSs were 3.5 ± 0.4 and 3.8 ± 0.4 for pre- and post-pubertal heifers, respectively. There was no statistical difference in an unpaired *t*-test in either BW (*P* = 0.38) or BCS (*P* = 0.18) between the heifer groups (Fortes et al., 2016).

After slaughter, tissue harvest was conducted as fast as possible to preserve RNA integrity. The entire liver was removed from the animal and three samples of 1 cm^3^ were dissected from the liver and snap frozen in liquid nitrogen. Samples were stored at -80°C until RNA extraction. In total, 12 liver samples were processed separately for RNA extraction and sequencing.

### Ribonucleic Acid Extraction

Total RNA was isolated from fragmented frozen liver tissue (∼100 mg) as previously described by Nguyen et al. (2017). Quality of the total RNA was evaluated using the RNA integrity number (RIN) measured with an Agilent Bioanalyzer 2100 (Agilent Technologies). Values of RIN range from 7.3 to 8.5, which indicated good quality of the RNA samples, which were sent to the University of California, Davis, for library preparation and sequencing.

### Library Preparation and Sequencing

mRNA was purified, fragmented, and used to synthesize cDNA, as described in (Cánovas et al., 2010). Briefly, after ligation of the adapters to the ends of double-stranded cDNA fragments, PCR was conducted to create the final cDNA library. Sequencing libraries were constructed with the TruSeq RNA sample preparation kit (Illumina, San Diego, CA, United States). RNA sequencing was conducted with a HiSeq 2000 Sequencer Analyzer (Illumina, San Diego, CA, United States). Quality control was performed using procedures described previously (Cánovas et al., 2013) using the application NGS quality control of CLC Bio Genomic workbench software (CLC Bio, Aarhus, Denmark). All samples passed all the parameters indicating a very good quality.

Sequence pair-end reads (100 bp) were assembled against the annotated bovine genome (release 77^1^). The “reads per kilo base per million mapped reads” (RPKM = total exon reads/mapped reads in millions × exon length in kb) was calculated for data normalization (Mortazavi et al., 2008). A threshold of RPKM ≥ 0.2 was utilized to annotated expressed genes (Wickramasinghe et al., 2012). Normalization and transformation data were performed using CLCBio Genomic workbench software (CLC Bio, Aarhus, Denmark) to transform the expression data from negative binomial distribution to normal distribution.

### Identification of Differentially Expressed Genes

Because genes with low counts can be easily biased without transformation, the base-2 log-transformed RPKM values were used. Mixed model equations are an optimal method for data normalization in gene expression studies (Reverter et al., 2005). We normalize the log-transformed RPKM values using mixed model equations to increase the sensitivity to detect differential expression and co-expression. This normalization approach for transformed RPKM values was previously described (Reverter et al., 2005; Cánovas et al., 2014). In more detail, differential gene expression after puberty was calculated using a mixed model: Y _*ijkpt*_ = μ + L_*i*_ + G_*j*_ + GAPT_*jkpt*_ + e_*ijkpt*_, where log 2-transformed RPKM (*Y_*ijkpt*_*) was modeled as a function of the fixed effect of the *i* library (*L_i_*) and of the random effects of gene (*G_j_*), and the interaction of gene × animal × physiological state × tissue (GAPT*_*jkpt*_*) for the *i* library (with 72 levels) and the *j* gene (with 16,978 levels) of the *k* animal (12 levels) in the *p* physiological state (with two levels) from the *t* tissue (with five levels). Finally, *e_*ijkpt*_* represents the random residual term. Our liver study was part of a larger experiment where five tissues were sampled per animal (hypothalamus, pituitary, liver, ovaries, and uterus), which allows fitting for tissue in the model. The VCE6 software^2^ was used to assemble and solve the mixed model equations and to estimate variance components associated with random effects. For each gene, the normalized mean expression was obtained based on adding the solutions G + GAPT. A *t*-test was used to test the hypothesis that the differential expression in post- vs. pre-pubertal heifers was significant. With the strict normalization performed, we then used *P* < 0.05 as the threshold to determine DE genes. This seemingly not very stringent nominal *P* was used in context with the strict normalization performed and the subsequent analyses, for which differential expression is one of many criteria under scrutiny.

### Identification of Key Gene Regulators

To determine gene regulators from the data, we mined the AnimalTFDB bovine database^3^, which comprises the classification and annotation of animal genomes for transcription factors (TFs), chromatin remodeling factors, and transcription co-factors. Among the annotated TF for *B. taurus*, 1,085 were expressed in the liver and further filtered for significance in terms of co-expression with DE genes, using regulatory impact factor (RIF) metrics (Hudson et al., 2009; Reverter et al., 2010). The RIF metric was explored in two measures: RIF1 and RIF2, calculated from the number of DE genes and the predicted interactions between TF and target DE genes (Reverter et al., 2010). In brief, RIF1 captured those TF that showed a large differential connectivity to highly abundant DE genes, whereas RIF2 focused on TF showing evidence as predictors of change in abundance of DE genes. A TF was considered as a key regulator if either of the two RIF scores was higher than 1.96 of the standard deviation, equivalent to a *P*-value level of at least 0.05.

### Gene Network Prediction

The partial correlation and information theory (PCIT) algorithm was utilized to detect the association between genes in a co-expression gene network (Reverter and Chan, 2008). In brief, PCIT explores all pair-wise direct and partial correlations among all possible trios of genes before identifying significant correlations that will establish edges during network reconstruction. The co-expression network predicted for the liver data was then visualized with Cytoscape (Shannon et al., 2003). From the large predicted network, we explored the subnetwork deemed to have biological significance for puberty trait. The subnetwork was used to identify the best trio TF that spanned most of the network topology with minimum redundancy. Specifically, an information lossless approach (Reverter and Fortes, 2013) that explored the 59,640 possible trios among 82 available TFs in the network was used to identify the best TF trio.

### Functional Enrichment Analysis

For enriched pathways and gene expression patterns, the Database for Annotation, Visualization, and Integrated Discovery (DAVID^4^) was utilized (Dennis et al., 2003; Huang da et al., 2009). In our study, the queried gene lists included genes and TF that formed the predicted gene network. This list of genes was utilized as a target gene list in comparison with a background gene list formed by all genes expressed in liver. Functional annotation chart revealed the most relevant (overrepresented) gene ontology terms and pathways associated with these gene lists, reporting an enrichment *P*-value for each annotation term. Significant results after Benjamini–Hochberg correction for multiple testing are reported.

## Results

### RNA-Seq Data and Normalization

The liver transcriptome data passed quality control performed with CLC Genomics workbench (CLC Bio, Aarhus, Denmark). Each individual sample had about 63 million sequence reads. Previous studies demonstrated that approximately 30 million reads are sufficient to detect more than 90% of annotated genes in mammalian genomes (Wang et al., 2011; Lee et al., 2013; Singh et al., 2017). The relatively high number of sequence reads generated in this transcriptome study indicates that our data are adequate for identification of DE genes. The number of unique reads and RPKM of each gene per physiological state are provided (Supplementary Table S1). Sequence data are available through the Functional Annotation of Animal Genomes project^5^.

### Identification of Differentially Expressed Genes

**FIGURE 1:**
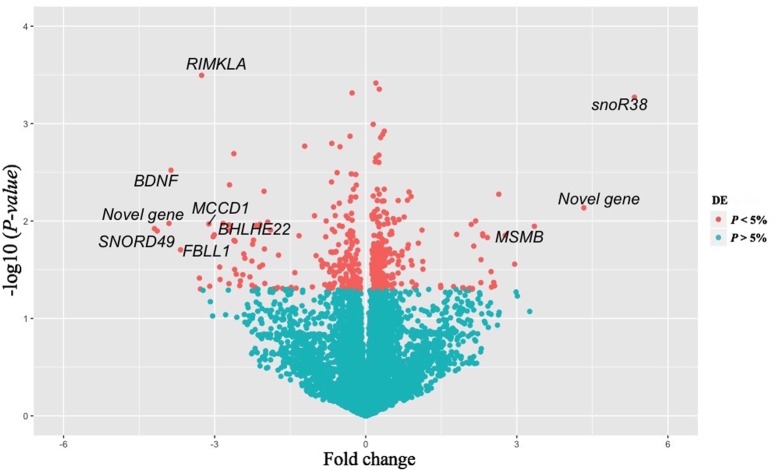
Volcano plot of differentially expressed genes (*N* = 452) in liver between pre- vs post-pubertal heifers. The *x*-axis represents the FC while the *y*-axis represents statistical significance for each gene. Red dots indicate genes that differ significantly (*P* < 0.05) between the two groups. Genes plotted in the left portion of the graph were expressed at a lower level in post-pubertal liver, and gene in the right-hand portion had higher expression levels post-puberty. Gene symbols are provided for genes with a |FC|≥ 3 and *P* ≤ 0.01.

A total of 16,978 transcripts were detected in both groups (pre- and post-puberty). A *t*-test of log-transformed data identified 452 DE genes (including 57 novel genes), of which 253 were up-regulated and 199 were down-regulated post-puberty (*P* < 0.05). Ten genes showed a threefold change (FC) difference in expression levels and *P* < 0.01 between pre- and post-puberty heifers (**Table 1**). **Figure 1** shows a volcano plot of log 2 FC vs. –log10 *P*-values for pre- vs. post-puberty gene expression. The gene annotation, FC, and *P*-value of 452 DE genes are presented in Supplementary Table S2. Significant DE genes were useful for understanding the biological mechanisms in the liver underlying puberty onset in Brahman cattle.

**Table 1:** The reads per kilobase per million (RPKM) mapped read values for genes that significantly differ in expression in liver between pre- vs. post-pubertal Brahman heifers (|FC|≥ 3, *P* ≤ 0.01).

ENSB tag^1^	Symbol^2^	RPKM_PRE^3^	RPKM_POST^4^	FC^5^
ENSBTAG00000043414	*snoR38*	1.415	6.750	5.335
ENSBTAG00000044882	*Novel gene*	0.546	4.877	4.330
ENSBTAG00000011660	*MSMB*	4.309	7.658	3.349
ENSBTAG00000042447	*SNORD49*	4.599	0.457	-4.142
ENSBTAG00000030124	*Novel gene*	6.815	2.908	-3.907
ENSBTAG00000008134	*BDNF*	5.890	2.022	-3.868
ENSBTAG00000017502	*RIMKLA*	6.187	2.928	-3.259
ENSBTAG00000045577	*MCCD1*	6.804	3.690	-3.114
ENSBTAG00000004657	*FBLL1*	4.978	1.947	-3.031
ENSBTAG00000033173	*BHLHE22*	6.871	3.865	-3.007

^1^*ENSB tag: Ensembl gene identifier according to www.ensembl.org. ^*2*^Gene: gene symbol related to the ENSB tag. ^*3*^RPKM_PRE: The RPKM in pre-pubertal heifers (average). ^*4*^RPKM_POST: The RPKM in post-pubertal heifers (average). ^*5*^FC: Fold change (RPKM _POST minus RPKM_PRE)*.

Insulin-like growth factor 1 is the major hormone secreted by the liver and is known to increase during puberty. In our study, the circulating IGF1 concentrations differed between pre- and post-pubertal heifers at euthanasia (*P* = 0.008) with the average serum IGF1 levels were 159.3 ± 25.5 ng/mL at pre-puberty and 203.2 ± 31.1 ng/mL at post-pubertal heifers. Although, RNA-seq analysis showed increase in *IGF-1* mRNA levels (2.01 ± 0.17 vs. 2.35 ± 0.19) after puberty in the liver, the result was not significant (*P* = 0.222).

### Identification of Key Gene Regulators

From AnimalTFDB Bovine database, we retrieved 1,085 TF that were expressed in the liver. Using RIF metrics, these known TFs were filtered for those most consistently associated with DE genes from this study, identifying 82 TF (*P* < 0.05). Supplementary Table S3 summarizes relevant data for these TF:RIF results, expression levels, and its description. Of the 82 TF, 19 genes (23%) coded for TF of the zinc finger family (ZNF). Further, out of the 82 TF, 2 TF (*SOX13* and *BHLHE22*) were themselves identified as DE genes.

### Predicted Gene Co-expression Network and Sub-network

**FIGURE 2:**
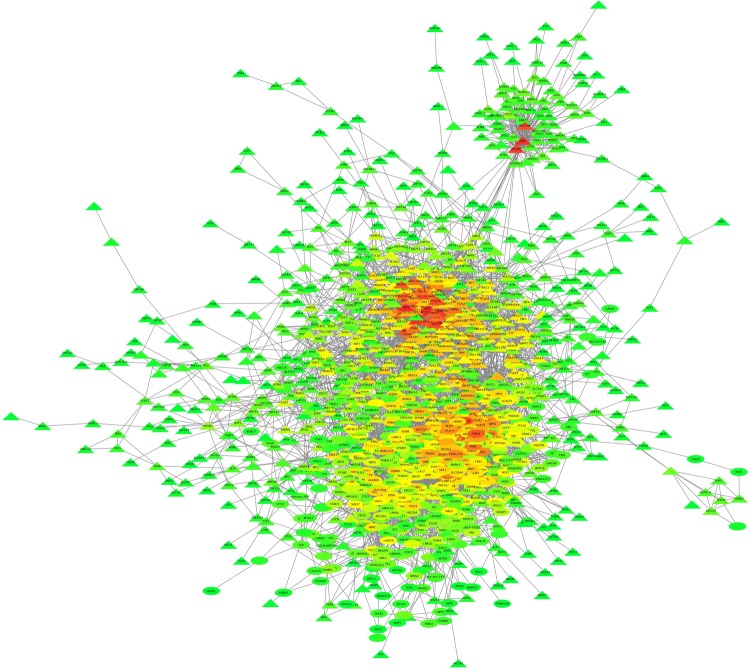
Liver gene co-expression network constructed by PCIT in pre- and post-puberty Brahman heifers. The entire network comprises 1,408 nodes (or genes) and 8,330 interactions. The color spectrum ranges from green to red for low and high number of connection, respectively.

**FIGURE 3:**
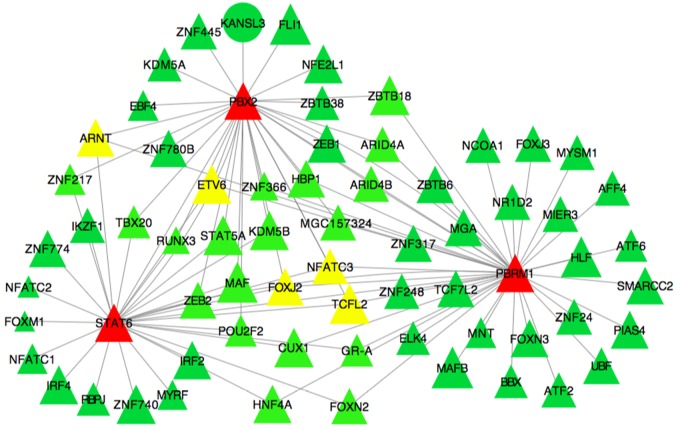
Subset of the liver co-expression network showing the best trio of TF and its predicted target genes in pre- and post-puberty Brahman heifers. Each node represents a gene. Nodes represented as triangles are TF and other coding sequences are represented as ellipses. Edges represent significant interaction between nodes. Node color indicates the number of connections of a specific node in the network. The color spectrum ranges from green to red for low and high number of connections.

Partial correlation and information theory algorithm determined significant partial correlations between DE and TF. A predicted gene co-expression network with 1,408 nodes representing genes and a total of 8,330 edges which account for the predicted interactions was constructed (**Figure 2**). In order to identify potential regulators of the predicted gene co-expression network, we focused on 82 TF contained in the network. After exploring all the TF trios, the top trio which spanned most of the network topology with highest connectivity (a total of 59,640 possible connections) and minimum redundancy was identified, including the signal transducer and activator of transcription 6 (*STAT6*), PBX homeobox 2 (*PBX2*), and polybromo 1 (*PBRM1*). **Figure 3** presents the connections between the top trio of TF and their potential targets.

### Functional Enrichment Analysis of Target Genes Involved in Gene Co-expression Network

Functional analysis using DAVID (Dennis et al., 2003; Huang da et al., 2009) allowed identification of biological functions overrepresented in our nodes in the co-expression network. Results showed that 91 GO terms (49.7%, 91/183) were significantly enriched in the biological process category, 8 GO terms (53.3%, 8/15) were significantly enriched in the molecular function category, and 21 GO terms (75%, 21/28) were significantly enriched in the cellular component category. In the biological process category, GO terms were related to liver development, gonad development, immune system development, and muscle organ development. Most importantly, many of the enriched GO terms were closely related to reproduction, including reproductive developmental process, reproductive structure development, and response to protein stimulus. In addition, the molecular function term associated with steroid hormone receptor activity was also enriched in our co-expression network. Pathway analyses revealed 10 significantly enriched pathways (47.6%, 10/21). Among these overrepresented pathways identified, we observed TGF-β signaling (adjusted *P* = 1.7 × 10^-4^) and Wnt signaling (adjusted *P* = 7.4 × 10^-4^) pathways. Supplementary Table S4 provides the full list of enriched GO terms and pathways, discovered using all genes in the network as the target dataset.

## Discussion

Reducing the age at puberty to increase cattle productivity is a major aim for *B. indicus* breeders. Although the hypothalamus–pituitary–ovarian axis is central to reproduction, the involment of the liver in controlling energy balance and affecting reproduction was reported before (Fontana and Della Torre, 2016; Montagner et al., 2016). IGF1 seems to be an important link between liver function and puberty onset (Akers et al., 2005). Although the post-pubertal liver samples were collected from animals with significantly higher progesterone levels, we found no direct evidence of increased synthesis of liver enzymes involved in the metabolism of steroid hormones. *IGF1* transcripts were not among the list of DE genes, although this endocrine signal from the liver is known to increase leading up to puberty and serum IGF1 was increased at post-pubertal Brahman heifers (current study). The bioavailability of and circulating half-life of IGF1 are determined by IGF-binding proteins (IGFBPs) and these may be an important consideration in puberty. It should be noted that most assays measure total IGF1, after extraction procedures that remove IGFBPs, like our results in the current study. Very few studies determine the small (<1% total), free bioactive fraction of IGF1 and/or concentrations of IGFBPs. Our results suggest that circulating IGF1 concentrations are influenced by multiple factors beyond *IGF1* gene expression.

In liver, 452 genes were DE between pre- and post-pubertal Brahman heifers (this study). Previously, 288 genes were DE between pre- and post-pubertal Brangus heifers (Cánovas et al., 2014). In Brangus heifers, liver DE genes contributed an abundant number of connections to the co-expression network and had the largest disappearance of connections after puberty. In short, network topology suggests that the liver warrants further scrutiny in Brangus heifers that was beyond the scope of the original publication (Cánovas et al., 2014). Here, we performed gene ontology and pathway enrichment analyses for both lists of DE genes, from the Brangus study and the current Brahman data. No ontologies were the same across breeds. Only 10 DE genes were the same across breeds and these are discussed further below. In short, biological differences between Brangus and Brahman heifers seem evident from the contrasting results in these transcriptomics studies.

The most DE genes (|FC| > 3 and *P* ≤ 0.01) in the liver of Brahman heifers were *snoR38*, *snorD49*, *MSMB*, *RIMKLA*, *MCCD1*, *FBLL1*, *BHLHE22*, brain-derived neurotrophic factor (*BDNF*), and two uncharacterized proteins (ENSBTAG00000044882 and ENSBTAG00000030124). These emerging candidate genes are discussed in the following paragraphs.

Small nucleolar RNA R38 (*snoR38*) and small nucleolar RNA SNORD49 (*snorD49*) are non-coding RNAs functioning in modifications of other small nuclear RNA (Matera et al., 2007). The snoRNA families are essential for major biological processes such as mRNA splicing and protein translation (Matera et al., 2007). There is limited evidence for the involvement of these snoRNA with puberty. The first deletion animal model of other snoRNA gene (*snorD116*) in mice revealed a role in growth and feeding regulation for the snoRNA family (Ding et al., 2008). The highest and lowest mRNA levels after puberty of *snoR38* (FC = 5.33) and *snorD49* (FC = -4.12) warrant further studies to understand the role that these non-coding RNAs play in liver function and puberty.

The gene β-microseminoprotein (*MSMB*) plays an important role in semen quality and fertilization (Anahí Franchi et al., 2008). Not restricted to male tissues, MSMB protein was also identified in porcine CL (Tanaka et al., 1995) and its gene expression was identified in human female reproductive tissues (Baijal-Gupta et al., 2000). Importantly, *MSMB* influences FSH secretion from pituitary gland, impacting on ovarian function (Thakur et al., 1981; Sheth et al., 1984; Frankenberg et al., 2011). It remains to be explored if liver production of *MSMB* achieves the pituitary signaling in growing heifers.

Very little is known about mitochondrial coiled-coil domain 1 (*MCCD1*) and fibrillarin-like 1 (*FBLL1*) function in the liver or with relation to puberty onset. One study in humans identified high expression levels of *MCCD1* in fetal liver (Semple et al., 2003). In cattle, *MCCD1* was DE in both RNA sequencing studies of puberty, ours, and the study by Cánovas et al. (2014). Its liver function merits further investigation.

The gene *RIMKLA* is involved in alanine, aspartate, and glutamate metabolism; as per KEEG pathway annotation (Kanehisa et al., 2017). Notably, glutamate and aspartate are major metabolic fuels for nutrient metabolism and oxidative defense (Yao et al., 2008; Brasse-Lagnel et al., 2009). It seems coherent that *RIMKLA* would be relevant for the liver metabolic function and perhaps it is another link between energy metabolism and reproduction to be explored.

The gene *BHLHE22* (FC = -3.00 and *P* ≤ 0.01) was revealed as the most down-regulated DE gene after puberty. This gene is also a significant TF (RIF2 score of -2.88). The *BHLHE22* gene belongs to basic helix–loop–helix (bHLH) family and plays significant role in cell proliferation and differentiation of several developmental pathways as well as cell fate determination (Lee et al., 1995; Ma et al., 1996; Farah et al., 2000; Xu et al., 2002). Further, an *in vitro* transfection assay used in mice showed that *BHLHB5* (an alias of *BHLHE22*) strongly inhibits the expression of the human *PAX6* promoter (Xu et al., 2002). The *PAX6* promoter is known as a TF involved in embryonic development and neurulation (Callaerts et al., 1997). A *PAX6* mutation was associated with isolated GH deficiency (Guerra-Junior et al., 2008). *BHLHE22* has been described as a transcriptional repressor of insulin expression in pancreatic β cells (Peyton et al., 1996; Melkman-Zehavi et al., 2011). Insulin can mediate follicular growth in cattle (Webb et al., 2004), stimulate GnRH release from the hypothalamus in combination with glucose (Arias et al., 1992), and may also facilitate IGF1 synthesis and secretion by the liver (Keisler and Lucy, 1996; Webb et al., 2004). The role of insulin in the regulation of lipid, glucose, protein homeostasis, and energy balance (Saltiel and Kahn, 2001; Liu and Barrett, 2002; Obici and Rossetti, 2003) suggests a link between insulin and the reproductive axis. In our study, *BHLHE22* was the most down-regulated gene, with lower expression in post-pubertal heifers (FC = -3.00 and *P* = 0.01). Lower expression of *BHLHE22* could mean decreased repression of insulin expression leading to increased GH and GnRH stimulus via IGF1 signaling. Therefore, liver produced *BHLHE22* could impact on animal growth and pubertal development.

The *BDNF* gene is related to neural development and peripheral metabolism (Binder and Scharfman, 2004; Pedersen et al., 2009). In the brain, *BDNF* can suppress GABAergic synaptic transmission by acute down-regulation of GABA_A_ receptors and thus can affect GnRH release (Henneberger et al., 2002). Previous studies suggested *BDNF* as a key component of the hypothalamic pathway controlling energy homeostasis and BW (Wisse and Schwartz, 2003; Xu et al., 2003; Jo and Chua, 2013). A genome-wide association studies (GWASs) in humans found *BDNF* to be related to timing of puberty and body mass index (Perry et al., 2014). Further, estrogen–BDNF–NPY has been noted as important tri-molecular cascade in understanding the hormonal regulation in hippocampus (Scharfman and MacLusky, 2006). It is unclear whether BDNF is able to cross the blood–brain barrier. Some researchers have found evidence for a link between central BDNF and peripheral BDNF (Poduslo and Curran, 1996; Pan et al., 1998; Rasmussen et al., 2009; Seifert et al., 2010), whereas others have argued that it does not cross the blood–brain barrier (Pardridge et al., 1998; Kyeremanteng et al., 2012). Hence, if further research can prove BDNF delivery across blood–brain barrier, it is possible that *BDNF* produced in the liver may have endocrine effects in the brain.

Brain-derived neurotrophic factor in the liver, similarly to skeletal muscle, results in increase of AMP-activated protein kinase (AMPK) and its downstream target acetyl coenzyme A carboxylase (ACC), inhibiting fatty acid synthesis and enhancing fatty acid oxidation (Matthews et al., 2009; Pedersen et al., 2009; Genzer et al., 2017). A study in humans suggested that fatty acid oxidation is higher in children than adults (Kostyak et al., 2007). Estrogen was also cited to regulate hepatic fatty acid oxidation (O’Sullivan, 2012). In liver, there is little information of precise mechanisms in which estrogen reduces fatty acid oxidation. Our study results led us to hypothesize that the interaction between estrogen and *BDNF* in fatty acid oxidation in liver, contributing to metabolic changes that can regulate puberty onset.

Comparing the liver transcriptional profile between our Brahman heifer study and a study by Cánovas et al. (2014) in Brangus heifers, we found 10 genes (including a novel gene) that were DE in both populations (*P* < 0.05). Five genes, *MCCD1*, *ADGRF2*, brain expressed X-linked 2 (*BEX2*), *PDZD7*, and *LRRC46*, had a | FC| > 1 in both breeds. The expression of these genes was up-regulated in Brangus heifers and down-regulated in Brahman heifers. The Brangus study involved eight heifers greatly differing in age and weight whereas our Brahman study used 12 heifers that were age and weight matched. Further, in the absence of a reference genome of *B. indicus*, we have utilized *B. taurus* reference genome for sequence assembly, and so the divergence between *B. taurus* and *B. indicus* genomes can impact our results. The significant difference in expression levels and patterns of these five DE genes between two breeds warrants further studies. The candidate gene *MCCD1* and its limited literature were discussed above. Similarly, *PDZD7* and *LRRC46* roles in puberty and liver function cannot be speculated from current knowledge. The remaining two genes, *ADGRF2* and *BEX2*, are discussed below.

The expression of adhesion G protein-coupled receptor F2 (*ADGRF2*, alias *GPR111*), in reproductive tissues and lung was reported (Fredriksson et al., 2002). Our study was the first to report mRNA expression of *ADGRF2* in the liver of *B. indicus* heifers. It is intriguing to suggest that *ADGRF2* could be another link between liver function and puberty, because G protein-coupled receptors have been associated with GnRH regulation (Noel and Kaiser, 2011).

The *BEX2* was observed as a DE gene in the ovary of pre- and post-pubertal Brahman heifers (Nguyen et al., 2017) and in the adipose tissue of pre- and post-pubertal Brangus heifers (Cánovas et al., 2014). The *BEX2* gene is highly expressed in the human embryonic brain and have a regulatory role in embryonic development (Han et al., 2005). A study of mice liver gene expression revealed a strong expression of *BEX2* in stem/progenitor cells (Ito et al., 2014). Further, *BEX2* is a downstream molecule of the mammalian target of rapamycin (mTOR) signaling pathway (Hu et al., 2015) that can regulate lipogenesis and ketogenesis in liver (Laplante and Sabatini, 2012). The mTOR pathway is also a known regulator of ovarian activity (Lu et al., 2017). In short, *BEX2* was DE in two studies of pubertal heifers, two different breeds, and thus it merits further investigation. It is possible that this is a liver signal with impact on ovarian activity.

Transcription factors play a key role in controlling gene expression, but their expression levels are often low and not detected as DE (Vaquerizas et al., 2009). The interactions between TF are important for tissue remodeling and temporal changes in gene expression (Ravasi et al., 2010). Differential expression analyses overlook vital changes in regulatory information. Adding an analysis focused on identifying key TF could help to understand the gene regulation processes under investigation (i.e., puberty). Previously, we found that TFs in the ZNF were DE and/or important TF in the transcriptomic profile of hypothalamus, pituitary gland, and ovaries in Brahman heifers undergoing puberty (Fortes et al., 2016; Nguyen et al., 2017). These studies noted that 26% of top ranking TF from hypothalamus, 28% from ovaries, and 22% from the pituitary gland top ranking TF coded for ZNF members in the same Brahman heifers (Fortes et al., 2016; Nguyen et al., 2017). Likewise, this current study revealed that 23% of TF identified by RIF analysis of liver transcriptome data belong to the ZNF.

The potential role of ZNF genes in the puberty process was suggested by several studies. A mouse study found that a mutation in regulator of sex-limitation (RSL), one of the Kruppel-associated box zinc finger proteins (KRAB-ZFP) genes, can impact reproduction by regulating expression patterns of target genes in liver (Krebs and Robins, 2010). In addition, ZNF genes have been implicated in the epigenetic control of transcription in the female primate hypothalamus around puberty (Lomniczi et al., 2015). Previous GWASs in women reported the association between single-nucleotide polymorphism located near *ZNF462* and *ZNF483* and age of menarche, which is the age of puberty in girls (Perry et al., 2009; Elks et al., 2010; Chen et al., 2012; Demerath et al., 2013). Expression of *ZNF127* was increased pre-puberty and decreased immediately before puberty (Abreu et al., 2013). Study of female monkeys also reported decrease of *ZNF573* mRNA levels in peripubertal animals (Lomniczi et al., 2015). Our study contributes to the growing body of evidence that support ZNF genes can influence puberty onset, a developmental role which may extend to tissues and organs outside of the reproductive axis.

In the sub-network, the trio of TF that spanned most of network topology with minimum redundancy and highest connectivity was *STAT6*, *PBX2*, and *PBRM1.* Previous evidence suggested these TF have important roles in liver and reproductive function. Specifically, the *STAT6* locus on BTA5 has been described as a QTL associated with reproduction (Kappes et al., 2000; Allan et al., 2009; Kim et al., 2009; Luna-Nevarez et al., 2011; Hawken et al., 2012). Further, this gene was identified as a key TF in a gene network constructed using GWAS results of first service conception in Brangus heifers (Fortes et al., 2012). Other studies noted the association between *STAT6* gene and age at first CL, an indicator of puberty onset in Brahman and Tropical Composite heifers (Fortes et al., 2010, 2011). Our study supported the potential role of *STAT6* in puberty onset in Brahman heifers.

The *PBX2* gene has a role in the development of ovarian follicles (Ota et al., 2008). Pbx2–Prep1 complexes repress HNF1α-mediated activation of the UDP glucuronosyltransferase family 2 member B17 (*UGT2B17*) promoter in liver cells (Gregory and Mackenzie, 2002). The *UGT2B17* gene, a sex steroid-metabolizing gene, has been associated with male infertility and impaired spermatogenesis (Plaseska-Karanfilska et al., 2012). Hepatocyte nuclear factor-1α (HNF-1α) is a homeodomain-containing TF that regulates liver-specific gene transcription (Mendel and Crabtree, 1991) and was suggested to control development and metabolism in a HNF-1α-null mouse study (Pontoglio et al., 1996). In liver, HNF-1α regulates the expression of glucocorticoid receptor (*GR*), *IGF1*, *STAT5*, and other GH-responsive genes (Lee et al., 1998; Lin et al., 2008). In our sub-network of predicted gene co-expression, *PBX2* and *STAT6* was also connected to *STAT5* (RIF2 score of 2.13) suggesting that the interaction between these TF could contribute to the regulation of growth, liver metabolism, and puberty onset.

Finally, the gene *PBRM1* seems to play a role in metabolic and immune system regulation, pertinent to liver expression. The gene *PBMR1* was described as a repressor of interleukin 10 (*IL-10*) transcription; an anti-inflammatory cytokine involved in metabolic syndrome (Mallat et al., 1999; Calcaterra et al., 2009; Wurster et al., 2012). Calcaterra et al. (2009) study showed high levels of IL-10 in serum samples of obese children. Further, IL-10 was proposed to be involved in the inflammatory network of metabolic syndrome in correlation with adiponectin (Böttner et al., 2004; Nishida et al., 2007). Of note, adiponectin plays a significant role in energy homeostasis (Lee and Shao, 2014). In liver, adiponectin can activate glucose transport as well as enhances insulin sensitivity (Berg et al., 2001; Combs et al., 2001; Ye and Scherer, 2013). A study of Holstein cows reported an association between follicular growth and the change in adiponectin and its receptor expression (Tabandeh et al., 2010). The role of *PBRM1* as a regulator of heifer puberty needs further investigation, but it is possible that it acts through adiponectin signaling.

After identification of DE genes and TF, GO and pathway analysis was performed to better understand the biological function of these genes in the context of puberty. Information about gene co-expression, enriched GO, and pathways facilitates the interpretation of RNA-Seq results. Based on GO analysis of 1,408 nodes from the liver co-expression network, multiple biological processes were affected. GO terms “reproductive developmental process” and “reproductive structure development” were significantly enriched and are logical in the context of puberty. Steroid hormone receptor activity and steroid binding were expected GO terms as liver is the principal site of steroid hormone metabolism.

We observed *TGF*-β *signaling* (*P* = 6.4 × 10^-6^) and *Wnt signaling* (*P* = 3.8 × 10^-5^) pathways among the enriched pathways. Both pathways were also enriched in pre- vs. post-pubertal results from the pituitary gland of Brahman heifers (Nguyen et al., 2017). Of note, transforming growth factor-beta (TGF-β) superfamily signaling plays a pivotal role in the regulation of cell differentiation, growth, morphogenesis, tissue homeostasis, and regeneration (Massague, 2012). In neural tissue, TGF-β1 one member of the TGF-β superfamily can increase GnRH gene expression as well as GnRH release (Prevot, 2002; Mahesh et al., 2006). Expression and release of GnRH are pivotal for puberty. The Wnt signaling pathway is an important physiological regulator of embryonic and liver development as well as mammalian hepatic metabolism (McLin et al., 2007; Marfil et al., 2010; Sethi and Vidal-Puig, 2010; Liu et al., 2011). Results from functional enrichment analyses provide evidence of pathways that are relevant for both liver metabolism and reproductive function. These pathways may point to some of the links between liver and reproductive function in *B. indicus* cattle.

We successfully exploited RNA-Seq data to identify the transcriptomic differences in liver between pre- and post-pubertal Brahman heifers. Previously, liver transcriptomics in *B. indicus* bulls and steers identified DE genes related to feed efficiency (Alexandre et al., 2015; Tizioto et al., 2015). This paper is the first attempt to demonstrate molecular mechanisms of puberty in liver of Brahman heifers. In the study, 452 DE genes were identified, many of which are closely related to reproductive developmental process, reproductive structure development, steroid hormone receptor activity, and steroid binding. In liver, TGF-β signaling and Wnt signaling genes may play a role in reproductive function. Moreover, the genes, *BDNF*, *STAT6*, *PBX2*, and *PBRM1*, might impact on the regulation of growth, liver metabolism, and puberty onset. As *BDNF* and estrogen can regulate fatty acid oxidation, we reasoned that *BDNF* and estrogen signaling may interact. This interaction can contribute to metabolic changes that can regulate the occurrence of puberty in Brahman heifers. Further studies are warranted to determine the function of these candidate genes. Our findings provide useful information for understanding molecular mechanisms in liver that may influence puberty onset of Brahman heifers.

## Author Contributions

LN performed RNA extraction, data analyses, interpretation of results and wrote the first draft. AR performed statistical analyses using mixed models. AC assemble, annotation and count of RNA sequencing data. MD and NC performed quality control of raw data. BV performed laboratory work. SA measured hormones. AI-T performed laboratory work. SL designed the experiment and interpreted the results. JM supervised library preparation and RNA sequencing. MT designed the experiment and interpreted the results. SM obtained funds for the research and supervised the project. MF performed the field trial, sample collection, experimental design, drafting of the manuscript, and interpretation of results.

## Conflict of Interest Statement

The authors declare that the research was conducted in the absence of any commercial or financial relationships that could be construed as a potential conflict of interest.

## Footnotes

1 ftp://ftp.ensembl.org/pub/release-77/genbank/bos_taurus/

2 ftp://ftp.tzv.fal.de/pub/vce6

3 http://bioinfo.life.hust.edu.cn/AnimalTFDB/download_index?tr=Bos_taurus

4 http://david.abcc.ncifcrf.gov

5 http://data.faang.org/home
